# Exploring the world of food with families: perspectives of low-income families on factors influencing their food choices

**DOI:** 10.1017/S136898002400020X

**Published:** 2024-01-18

**Authors:** Elena Vaughan, Eleni Spyreli, Michelle McKinley, Marita Hennessy, Jayne Woodside, Colette Kelly

**Affiliations:** 1 Health Promotion Research Centre, College of Medicine, Nursing and Health Sciences, University of Galway, University Road, Galway, Republic of Ireland; 2 Centre for Public Health, School of Medicine, Dentistry and Biomedical Sciences, Queens University, Belfast, Northern Ireland; 3 INFANT Centre, University College Cork, Cork, Republic of Ireland

**Keywords:** Food choices, Families, Low income, Food poverty, Bronfenbrenner’s Ecological Systems Theory

## Abstract

**Objective:**

The aim of this study was to investigate the social and environmental factors involved in the food decision-making processes of families living on lower incomes on the Island of Ireland.

**Design:**

A qualitative design was employed for this study, using photovoice and creative mapping methods. Parents were requested to take photos and draw maps of their food environments. Interviews were then conducted with parents, using the materials produced by parents as a cue to discuss their food environments, influences and decision-making processes around food choices.

**Setting:**

The participants were interviewed online via Microsoft Teams.

**Participants:**

The participants were parents or guardians of children between the ages of 2 and 18 who self-defined as ‘living on a tight budget’.

**Results:**

Twenty-eight participants were recruited and interviewed for this study, including twelve parents in Northern Ireland and sixteen in the Republic of Ireland. The findings were mapped on to Bronfenbrenner’s Ecological Systems Theory and showed that multiple, overlapping and intersecting factors at the individual, micro-, meso-, exo-, macro- and chrono-system were implicated in family food choices. Upstream factors in particular, including structural, policy and commercial determinants, appear to be significant drivers of behaviour.

**Conclusions:**

While the findings suggest that a complex range of factors are involved in family food choices, it is clear that policy measures and regulations are needed to stave off the impacts of rising social inequality and food poverty. Health promoters should strive to find non-stigmatising interventions to bridge the nutritional divide experienced by lower-income families.

The link between dietary behaviours and health is well established, and poor nutrition is a leading risk factor for morbidity and mortality worldwide^(1)^. Socio-economic status is one of the most salient determinants of access to a diet of higher nutritional quality, and a clear social gradient can be observed^(2)^. Children and adolescents from higher socio-economic backgrounds are more likely to consume fruit and vegetables daily and more likely to meet nutritional guidelines than their peers from lower socio-economic backgrounds^(3)^. As such, food poverty – defined as the ‘inability to afford or have reasonable access to a nutritionally adequate diet^(4)^’ – is an acknowledged social determinant of health and is associated with significant adverse health outcomes. Food poverty has been linked to negative mental health outcomes, as well as diabetes, hypertension and higher odds of chronic disease amongst adults^(5)^; and with cognitive problems, anaemia, aggression and anxiety, higher risk of hospitalisation and poorer mental, oral and general health among children^(6)^.

While external structural factors such as socio-economic status are key determinants of health and diet, dietary behaviours and food choices are shaped and constrained by an additional intersecting array of factors at the individual, environmental and societal level^(7)^. Other external macro-level factors include commercial and corporate drivers^(8)^, economic factors^(9)^, cultural factors^(10)^ and policy and political considerations^(11)^. Community-level environmental factors are also salient^(12)^, including type, availability and accessibility of food outlets and stores^(13)^. Other determinants of dietary behaviours include individual-level factors, such as sex or gender^(14)^; age^(15)^; family-level characteristics and dynamics, such as family structure^(16)^.

Within the family, parents play a significant role in developing children’s food choices, and eating habits acquired in the home environment can remain consistent across their lifespan^(17)^. There is evidence that eating together as a family and parental role modelling are associated with eating behaviours^(18)^ and with food acceptance^(19)^. Regular family meals positively influence child and adolescent dietary habits both in the short and long term^(20)^ and may reduce the risk of obesity^(21)^. More recently, the role of family mealtimes on well-being, family communication and child socialisation has gained interest^(22)^. The causal mechanisms for the family setting and healthy dietary habits are unknown but socio-cultural, demographic and environmental factors likely play a role.

In the Republic of Ireland (ROI), approximately 9 % of the population are estimated to be in food poverty^(23)^, while in Northern Ireland (NI), 7 % of households are classified as food insecure^(24)^. Low-income groups spend a greater proportion of their weekly budget on food, estimated at up to 32% of net income to purchase a healthy weekly food basket^(25)^. With a growing cost of living crisis triggered by the inflationary impacts of COVID-19^(26)^, and the on-going threat of climate change to global food security^(27)^, it is likely that food poverty will affect a significantly greater number of families on the Island of Ireland in the near future. Indeed, there is evidence to suggest that households are increasingly being affected, with 51% of respondents (*n*=1130) in a recent survey in ROI cutting spending on fuel and other household expenditures to afford food^(28)^. Similarly, in NI, there has been a more than threefold increase in food-bank use since 2015^(29)^.

While parents can ultimately shape their children’s preferences and long-term eating behaviours, parents themselves are influenced, and often constrained, by various factors when deciding what, where and when to eat with their children. This study sought to understand the competing and interacting factors affecting family food choice, with a particular focus on social and environmental influences, and how parents on a low income strategise to make daily decisions about food. Given the increasing economic challenges faced by families in Ireland, as elsewhere, understanding these decision-making processes has implications for designing and implementing effective health promotion interventions that mitigate against the impacts of food poverty.

## Methods

This study was part of a larger project aimed at assessing the evidence and gathering data on the factors that influence daily family food-related decisions of families on the Island of Ireland^(30,31)^. A qualitative approach was used for this study, involving the use of creative mapping and photovoice methods with semi-structured interviews to collect data from research participants. Two separate study teams collected data from parents in NI and ROI between July 2020 and February 2021. Initial plans to collect data in-person were curtailed by COVID-19 restrictions; consequently, data collection pivoted online. This section outlines selection of participants, data collection methods and analytical techniques.

### Patient and public involvement

An integrated knowledge translation approach was adopted to this study, applying the principles of public and patient involvement^(32)^. A panel of parents was recruited through extant networks, parent groups and via social media to advise on aspects of the study including recruitment, data collection and dissemination. The panel provided advice on appropriate and sensitive language to use in recruitment and study materials. For example, on the advice of the panel, we were advised against formally assessing income and to use the term ‘living on a tight budget’ rather than ‘low income’. Parents on the panel were recompensed for their time.

### Sampling and recruitment

A sampling matrix was employed to ensure a diverse cohort of family types, including parents of both older and younger children, urban and rural dwelling families and single and two-parent households. Participants were recruited using advertisements (e.g. email, graphics/digital fliers and WhatsApp messages) via health promotion networks and organisations, community groups, social media, parent groups and family resource centres. A €40/£35 voucher was offered as recompense for their time. Participants were eligible if they were parents or guardians of children between the ages of 2 and 18, and if they self-defined as ‘living on a tight budget’. All participants were provided with information sheets and gave written informed consent via email. Ethical approval was granted from two university research ethics committees.

### Data collection

Photovoice and creative mapping methods were used as part of the data collection process. A strength of the photovoice^(33)^ and creative mapping methods^(34)^ is their effectiveness in revealing real-life experiences. As participatory approaches, these methods can empower individuals to engage in the research process in a creative and personal, yet agential manner, reducing the power differential between researcher and participant. Consequently, they are ideal methods for glimpsing into the private domestic lives of families, of which a very fundamental component is the feeding and nourishing of one’s family members.

Data were collected and analysed by two researchers (EV & ES), with 17 years of combined experience in qualitative research methods. As scholars working in the disciplines of health promotion (EV and CK) and public health (MH, MMK and JW), our work is focused on shedding light on the wider social and structural determinants of health; consequently, we agree, with Schrecker (2016), that the task of addressing health inequalities requires scholarship ‘capable of identifying the relevant macro-micro connections^(35)^’.

In an initial brief phone/Microsoft Teams™ interview, participant demographics were captured using a brief questionnaire, and data collection methods were explained. Participants were asked to draw maps of their food environments and to take photos of any food-related activities (e.g. food shopping, receipts, advertisements and food preparation), with guidance given to not include identifying images of individuals. Pictures and maps were sent to the research team via email or WhatsApp, uploaded to a secure server and subsequently deleted from phones/hard-drives. Following this, participants were interviewed over Microsoft Teams™ using a semi-structured guide aligned to the photovoice method (see online supplementary material, Supplemental File 1). Photos and the maps were displayed during the video-call on PowerPoint to use as a reference point for discussions around their family’s food environment (see online supplementary material). Interviews were recorded and automatically transcribed in Teams. Transcripts were reviewed for accuracy, cleaned, edited and anonymised before being uploaded to NVivo™ for qualitative content analysis^(35)^.

### Data analysis

Initially, an inductive approach was taken to data coding. This was conducted separately by (ES) in NI and (EV) in ROI before being shared. Following a first round of coding, researchers compared codes and preliminary themes, critically and reflexively discussed the relevance of findings, and commonalities and differences in both samples. During discussions, it was agreed among the research teams that the findings appeared to map well on to Bronfenbrenner’s Ecological Systems Theory (BEST)^(36)^, and that this model would be an appropriate conceptual lens through which to illustrate the findings.

### Illustrating the findings

**Fig. 1:**
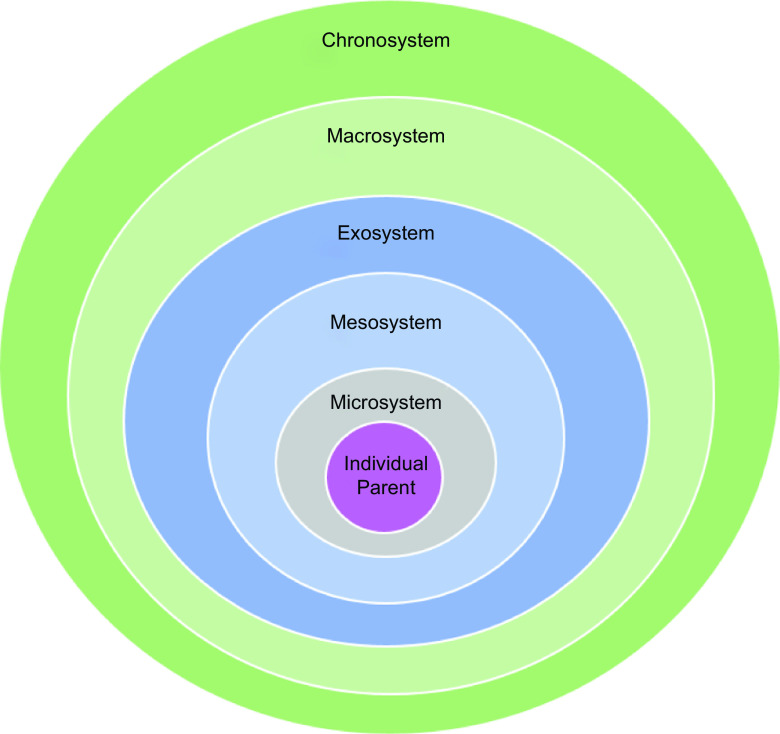
Bronfenbrenner’s Ecological System’s Theory Model based on: Bronfenbrenner, U. (1979). The ecology of human development: Experiments by nature and design. Cambridge, MA: Harvard University Press. [Graphic elaboration author’s own]

The findings are presented in alignment with BEST (see Fig.1). Bronfenbrenner’s Ecological Systems Theory consists of five distinct but inter-related spheres that describe interactions that shape behaviours in the social field at individual, organisational and policy levels across the life-course. Originally conceived of as an explanatory model to account for the range of social and structural determinants of child development, BEST is used across a variety of disciplines and is a helpful framework for conceptually separating out the distinct but inter-related spheres of social and environmental influence on an individual’s actions, behaviours and outcomes. Each of the five layers, plus individual-level characteristics, is outlined in Table 2 alongside the corresponding family food factors identified during the course of the analysis. The results section is structured accordingly, with consideration of each ecological systems level and the family food factors that correspond with these spheres of influence; illustrative pseudonymised quotes are provided throughout.

## Results

A total of twenty-eight participants were recruited for the study, with interviews carried out with twelve parents in NI and sixteen parents in the ROI. No one refused to participate or dropped out of the study. Table 1 shows characteristics of the sample, including information on level of education, employment status and marital status. Some differences were observed between the samples in NI and the ROI; half (50%) of the sample from the ROI were in full-time employment, while in NI the majority of participants had full-time domestic duties (58%). Parents with children under 12 were also disproportionately represented in both samples (57%) and only two men, one each from NI and ROI participated. Most of the participants were educated to degree level (68%). Information on income level was not sought, however, as eligibility for benefits is contingent on income, this was used as a proxy; the majority of participants (79%) in the sample were eligible for state benefits, a medical card or a general practitioner (GP) visit card. The median age of participants was 41·5 years (sd 6·8).

**Table 1:** Participant details and characteristics

Demographic characteristic	Response	*n* ROI	% ROI	*n* NI	% NI	*n* IOI	% IOI
**Sex**	Female	**15**	94 %	11	92 %	26	93 %
	Male	1	6 %	1	8 %	2	7 %
Place of birth	ROI/NI	11	69 %	7	58 %	18	64 %
	Other UK	1	6 %	3	25 %	4	14 %
	Poland	1	6 %	1	8 %	2	7 %
	Ukraine	1	6 %	0	0 %	1	4 %
	United States	1	6 %	0	0 %	1	4 %
	Germany	1	6 %	0	0 %	1	4 %
	Vietnam	0	0 %	1	8 %	1	4 %
Ethnicity	White	16	100 %	11	92 %	27	96 %
	Asian	0	0 %	1	8 %	1	4 %
Education level	Secondary	2	13 %	1	8 %	3	11 %
	Certificate or diploma	4	25 %	2	17 %	6	21 %
	Degree	6	38 %	6	50 %	12	43 %
	Postgraduate	4	25 %	3	25 %	7	25 %
Employment status	Part-time employed	3	19 %	2	17 %	5	18 %
	Full-time employed	8	50 %	2	17 %	10	36 %
	Full-time domestic duties	2	13 %	7	58 %	9	32 %
	Disability	2	13 %	0	0 %	2	7 %
	Student	1	6 %	0	0 %	1	4 %
	Retired	0	0 %	1	8 %	1	4 %
Marital status	Married or living with partner	9	56 %	6	50 %	15	54 %
	Divorced or separated	1	6 %	5	42 %	6	21 %
	Single	6	38 %	1	8 %	7	25 %
Eligibility for benefits	Yes	13	81 %	9	75 %	22	79 %
	No	3	19 %	3	25 %	6	21 %
Children’s ages	Under 12	10	63 %	6	50 %	16	57 %
	Under and over 12	1	6 %	4	33 %	5	18 %
	Over 12	5	31 %	2	17 %	7	25 %

ROI, Republic of Ireland; NI, Northern Ireland; IoI , Island of Ireland (Republic of Ireland and Northern Ireland).

**Table 2:** Ecological systems levels, with relevant system features and corresponding family food factors identified in this study

Ecological Systems level	Relevant system features/influences	Corresponding family food factors
Individual	Sex/gender Health status	Individual health status and/or dietary needs of family members
Microsystem	Family Friends Peers	Family composition Family support Friend/peer influences Parents knowledge/skills/information about food
Mesosystem	School Neighbourhood Local context Workplace	Child-minders/schools/after-schools Rural/urban location Proximity of amenities
Exosystem	Social and mass media Policy/political environment Education system Parents’ economic situation Corporate influences Other external influences	Use of apps, social media and other online resources Family budget constraints Availability of school meals Marketing and store offers/meal deals Benefit payments and other government assistance programmes Parents’ working practices and availability of time
Macrosystem	Cultural beliefs and values Ideologies	Societal and cultural beliefs and values around food Traditions
Chronosystem	Socio-historical events and environmental changes over time	Impact of COVID-19 pandemic

### Individual-level factors: Balancing child preferences with parental responsibility to meet dietary requirements

At the individual level, family food choices were driven largely by the needs and preferences of children across different developmental stages. Parents were cognisant of the need to ensure a healthy and balanced diet for their children to maintain healthy growth and development. Purchase and preparation of fresh food, home cooked meals and consumption of fruit and vegetables, dairy products and meat were regarded as important:

So as long as I know he had some fruit or something, fruit or veg in the day makes me a wee bit more content (Niamh, NI).

Parents perceived ‘fussy’ eating to be a barrier to ensuring children consumed healthy diets. Differences in preferences were acknowledged at different developmental stages, with younger children especially having to be coaxed or coerced into eating vegetables. Strategies used by parents to overcome these barriers included disguising vegetables in meals, modelling behaviours and direct enforcement:

My son, he’s just so awkward and fussy … He eats … pizza or pasta with red tomato sauce in it that his dad makes with … a lot of vegetables mixed into it and blended down (Angela, NI).

Several parents alluded to their adolescent children becoming more health and diet conscious as they got into their teenage years and how this mediated their dietary needs and preferences:

He’s six foot four and he plays a lot of sport so he’s always hungry and always in the gym. That was actually just one of his meals – sweet pepper with fajita turkey breast, and low fat cheese, because he is also calorie watching (Theresa, ROI)

### Microsystem factors: Support and influence of friends and family

The microsystem is the first level of BEST and includes the immediate relationships in one’s life. Friends, neighbours and family were identified as micro-system factors that mediated food decision-making. Parents described how they were influenced by friendships in the local community, with many exchanging recipes and suggesting dishes. Similarly, parents noted the influence of children’s friends and how interactions with their families introduced them to new dishes or foods:

If she was like staying over at a friend’s house and say they had something different at dinner or lunch or whatever, she’d come home and she’d want me to try and make it (Sarah, NI).

Extended family were noted as an important source of support and resources, with food occasionally gifted and shared, especially when budgets would not quite stretch far enough, and during lockdowns when things may have been especially tight:

My cousin and my family are helping me a lot during this lockdown. She is in the disadvantaged area … so she receives the food packages, huge boxes at home, and she gave them to me (Katarina, ROI)

### Mesosytem factors: Schools as sites of importance, and influence of local environmental context

The mesosystem relates to interactions between the immediate contacts and other external relationships. Schools, pre-schools and local childcare facilities were sites of importance in the day to day food environments of children. Child-minders, crèche facilities, after-school facilities and breakfast clubs were mentioned frequently – particularly by working parents – as places outside the home where children would be fed, often on a subsidised basis, during the week:

There is a school canteen and then they get, like, she can get pasta and she get chicken goujons, and it’s pretty good. And it’s very reasonable. It’s only €1·50 (Mary, ROI)

In NI parents who qualified for free school meals expressed relief, as they did not have to worry about their children’s nutrition during school hours, even in cases where these meals were not of equal quality to those cooked at home. Most schools in the ROI do not provide school meals except in schools designated as disadvantaged (discussed below), so packed lunches are the norm for most. Parents described that a considerable proportion of their shopping had to cover school lunches and snacks, ensuring a variety of items that children would eat. Meals were, however, often provided in after-schools and crèches, where there were indications of the mediating influence of socialisation with school/crèche peers:

It’s so funny because [in crèche] they’ll eat fish and stuff they won’t even eat at home, but when they see their friends, they’re not fussy. (Stephanie, ROI)

The immediate environmental and local context, such as proximity and availability of shops, restaurants and amenities were also salient aspects that influenced food choices. Most participants reported a good variety of options of places to shop for food, even in rural areas. Choice of which store to use often then revolved in part around other factors such as availability of parking or public transport links, accessibility of location and perceived convenience or hassle:

There’s a fruit and veg kind of stalls on Thursday and Friday in [name of village], but just parking wise, I don’t go there. (Karen, ROI)

Choice was not necessarily overly limited by living in a rural location albeit travel distances to and from shops were greater. This meant a greater reliance on having a car and thus incurred additional time and cost associated with purchasing food:

No you can’t [be without a car]. I’m three kilometres away from the [food bank], six kilometres away from [the supermarkets]. And there’s no public transport out here. (Sally, ROI)

### Exosystem factors: Impact of upstream factors and corporate environment, and time as a limited resource

The exosystem refers to organisations and social structures external to the individual that operate independently of them. Relevant here were external up-stream economic and policy factors, such as the price of food and availability of benefits, the commercial and digital environment, and time available in the working day.

Available food budgets for families were determined by economic factors, employment status, income levels and eligibility for benefits. Staying ‘within budget’ required skills to plan and manage family finances. Food costs were noted as a barrier to choosing healthier options, and that if prices were lower, people would be ‘happier to have vegetables’ (Alice, NI). Those who were in receipt of benefits and subsidies spoke to the importance of these to their budget and how, in particular, monthly payments such as children’s allowance (ROI) provided a necessary boost to buy essentials for the month:

The start of every month would be a major factor in my shopping because I would get the child benefit, which means my budget for the week would be increased. So I buy staples for the cupboard, which would include rice, a bag of potatoes and something that’ll last. (Sally, ROI)

Tight budgets meant that families needed to know how to navigate the commercial environment. Participants described store architecture and product placement as tactics that budget-conscious shoppers needed to be wary of. One marketing strategy observed was that ‘cheaper things are at the bottom’ (Sarah, NI), while more expensive items are placed at eye-level or highlighted with brighter colours. To keep within budget, most families relied on special offers and vouchers when doing the weekly food shop. Meal plans and weekly shops were often planned around the availability of such offers and this was a key driver of purchasing behaviour. Of note, and indicative of the growing influence of digital marketing, many parents referenced the use of store apps when doing the weekly shop or meal planning:

The key thing here is that [name of store] have introduced this app where you get different items reduced, or free items etc. So that’s kind of a driver. (Shane, ROI)

Digital food marketing was also raised by participants, many of whom took screenshots of their social media feeds to show the type of content that appeared, or people, sites and groups they followed. Types of advertising included sponsored or branded native content, while participants additionally spoke frequently about getting ideas from celebrity chefs, fitness, and lifestyle influencers for food purchasing and preparation:

I’m on Instagram a lot. That’s like my favourite thing to browse, and I will take screenshots at least once a week of things I want to try. (Siobhan, ROI)

Time was an especially scarce commodity and an influential factor in the choices of single and working parents in particular. Shopping trips would need to be planned around school/crèche pick-up times and work hours, which meant convenience was prioritised. Lack of time was implicated in the use of convenience and processed foods on busy days:

I’ve so little time that I would just have like a sandwich for dinner, and a cup of tea or something. So sometimes in the mid-week I would make for [my daughter] like fish fingers, a waffle and maybe spaghetti hoops. So it’s not in any way nutritious. (Karen, ROI)

### Macrosystem factors: Influence of parental values, beliefs and cultural background

Macrosystem factors included values and beliefs around food, cultural differences in educating children about food, and the acceptability of free school meals. Some participants expressed strong views about the value and importance of meal time as a time for family. For these parents, cooking ‘good’ food and making meal time an occasion was seen as an integral part of being a family:

Meal time would be a very important part of the life, to come together and sit together. And prepare the table with candles, especially in winter or in autumn and spring time. It’s important to have a candle on the table and to make a nice atmosphere. (Sylvia, ROI)

Well we always eat together. It’s something that I was brought up with, where, you know, family time is dinner time. (Emma, NI)

Family meal times were important, not just for family bonding, but also as an opportunity to contribute to child development by sharing traditions and culture, and learning about healthy eating and food preparation as a life-skill. Participants alluded to beliefs about their responsibility to feed children healthy food and to pass on knowledge about cooking and nutrition:

I want [my daughter] to know how to cook. Just to have the life skill and for her to enjoy it. I think it’s an important skill to have for the future. (Karen, ROI)

For parents constrained in their ability to fulfil this role because of barriers related to finances, time or lack of knowledge and skills, there was a sense of guilt:

I suppose I’d have a bit of guilt about it - that I’m not feeding the kids properly. But this is all my stuff … it’s just my issues. (Lynda, ROI)

Cultural differences were noted in respect of attitudes and beliefs around food. One woman observed differences between Ukraine and Ireland in regards to attitudes and beliefs around free school meals, saying she believed the concept to be somewhat stigmatised in Ireland, whereas in Ukraine it was viewed as an appropriate use of taxes. Similarly, another participant observed differences between Ireland and Germany in approaches to educating children about food, where she observed German children were encouraged to be more independent, try new foods, and learn cooking skills from a younger age. For Polish and Vietnamese participants, cooking food native to their homelands was important in passing on that culture and tradition to their Northern/Irish children. Perhaps reflecting the legacy of the Famine in Ireland, participants who were born and reared in Ireland spoke about being raised to be highly conscious of food waste and were often preoccupied with avoiding it:

I was brought up on no waste, so as little waste as possible (Rachael, ROI)

The [picture of] apples is just an example then of food waste - It’s a pet hate of my wife. (Shane, ROI)

### Chronosystem factors: Impact of COVID-19

As a result of COVID-19 and the ensuing health protection restrictions, families reported changes in food-purchasing, preparing, and consumption practices. Many parents reported using more online ordering and home delivery services. Most commonly reported were changes to the frequency with which they did their food shopping. This was driven by fear of infection, and also reflected the strict nature of Ireland’s COVID-19 measures:

I was doing it every second week … I don’t like shopping. I find it stressful at times. And especially during lockdown, when, you know, you’ve got your traffic light system and you’re trying to stay away from people (Anna, ROI)

Almost all families reported increases in food consumption and snacking, mainly involving less healthy high sugar, high-fat foods and drinks. There were also reported increases in consumption of takeaways. Boredom, comfort and convenience were commonly cited reasons:

I’ve been in survival mode during COVID … Since COVID it’s definitely more processed food than we would have eaten. (Dave, NI)

Many parents also observed changes in their spending on food. Parents on a very tight budget who regularly relied on store deals and special offers were often obliged to take extra time to plan meals, using store apps and advertisements to see what was on offer a day ahead of doing the weekly or fortnightly shop. While some found their overall outgoings had decreased because of fewer opportunities to buy impromptu purchases, spending on food in the home more often than not increased, even where budgets were particularly tight:

Definitely since lockdown … just I suppose the constant breakfast, lunch and dinner, as well as the snacks in between. And it just depleted the budget, I suppose, because my budget would be €35 max, including nappies and food for a week. (Sally, ROI)

The COVID-19 crisis implied hardship for some, however, for others there were some silver linings. These included a growing appreciation and consciousness of food, having more time to spend and eat meals together as a family, and getting the children more involved in food preparation:

The kids help out a lot more during lockdown. We kind of encourage the elder two to pick out recipes they wanted to make. And then I would get the stuff they needed in the shop. (Shane, ROI)

## Discussion

The overall aim of this study was to explore the factors and contexts that influence daily family food related decisions, and how families navigate and use strategies to make food decisions within their food environments. In line with other studies, children’s needs and preferences, time, finances, and availability and accessibility of grocery stores were among the salient factors^(31)^. Analysis of these factors through the framework of BEST, however, shows an altogether more complex picture of family food decision-making. It is clear that the factors implicated in family food choices are not disconnected from each other, but interact with, and are contingent on factors at other levels. For instance, while on an individual-level parents were knowledgeable about their children’s nutritional and developmental needs, and expressed a sense of responsibility to ensure they were met, they were often constrained in their ability to do so by upstream structural factors.

These upstream factors included lack of time in the working day (due to labour practices, school times and shop opening hours), the local environmental context (including access to and availability of transportation), and availability of and eligibility for school meals and benefits (governed by national-level policy). Such findings demonstrate the importance of structural and policy measures in mediating family food decision-making processes. For instance, considerable differences were apparent between NI and ROI where national-level policies that determine eligibility for or availability of free school meals vary substantially. Subsidised or free school meals are the norm in NI for those on low income, while in ROI they are only available in specially designated schools rather than based on individual need. Particularly in the context of growing food poverty, the lack of free school meals for those on lower incomes in ROI may have negative implications both for child obesity levels and educational attainment, which in turn could exacerbate existing social and health inequalities^(37)^.

The importance of policy measures in mitigating such health inequalities was apparent in this study. Parents on particularly low incomes or who had a greater reliance on social welfare described how universal child benefit was vital in helping to plug gaps in food budgets. Some were nevertheless additionally reliant on food banks, food donations and other similar initiatives to supplement their diets, particularly during the COVID-19 crisis. For such parents, it may be inaccurate to talk about food decision-making processes per se, as that implies a greater amount of choice than most have. Some expressed a sense of stigma and guilt associated, respectively, with needing assistance and with their perceived failure to consistently provide their family with healthy nutritious foods to meet their dietary needs. Inasmuch as this is a concern because of the negative impact of stigma on individual health outcomes^(38)^, it is further suggestive of the role of stigma in the syndemic of food insecurity and diet-related chronic diseases^(39)^ and warrants further investigation.

Other upstream factors included the commercial environment, the influence of which was readily apparent. These findings align with recently articulated conceptualisations of the commercial determinants of health (CDoH), which define commercial determinants of health as the ‘systems, practices and pathways though which commercial actors drive health and equity^(8)^’. For instance, while parents acknowledged the potential for store architecture to drive less healthy choices and impromptu purchases^(40)^, the time, convenience and bargains afforded by large national and multi-national stores was a huge draw. This was especially the case for working parents, for whom lack of time due to conventional labour practices – another component of the commercial determinants of health – was an additional barrier to healthful eating^(41)^. The impact of social media and digital food marketing (DFM) on parents’ decision-making processes calls for further attention. There is evidence to suggest that parasocial interactions with social media influencers lead to positive attitudes to brands and intentions to buy^(42)^, as well as promoting impulse purchases^(43)^. Parents may additionally lack awareness and understanding of how DFM^(44)^ and social media more broadly^(45)^ are operationalised to increase product recall and recognition and thus drive unhealthy choices.

Finally, the findings show the COVID-19 crisis had an enormous impact on patterns of food purchasing and consumption behaviours. Those who lost employment due to COVID-19 reported a greater reliance on welfare benefits and subsidised food sources, resulting in greater food insecurity^(46)^. Parents reported an increase in bulk-buying, batch cooking and freezing to stretch supplies; however, they also reported an increase in unhealthy eating behaviours, which may have longer term implications for health outcomes^(47)^. More positively, the greater involvement of children and adolescents in food preparation may have had benefits for diet quality^(48)^, while the increased opportunities for family meal participation are also associated with a more favourable diet^(49)^.

## Strengths and limitations

Male parents, ethnic and racial minorities were under-represented in this study. Future studies would be advised to devise additional recruitment techniques to attract a more ethnically and gender diverse sample. It is possible that by relying on participants who self-identified as ‘living on a tight budget’ individuals who would not be categorised as ‘lower income’ based on socio-economic criteria may have self-selected. However, given that the majority of the sample were eligible for benefits, we do not believe this unduly negatively affected recruitment or study objectives. Indeed, we believe the involvement and advice of the parent panel was a strength which helped inform the recruitment methods and ensured study materials were apposite and relevant. A further strength of this study was the use of creative research tools which were used to fully engage participants in the study. While unplanned for, having to pivot online meant that we were able to recruit a diverse sample of parents from across a wider geographical area than originally intended.

## Conclusion

Navigating the social, economic, political and commercial environment to ensure families are adequately nourished is becoming more challenging for many, particularly for families on lower incomes. The findings here suggest that upstream policy measures strongly mediate access to and availability of healthful nutritious food. While this includes more obvious policy measures such as school meals and welfare benefits, it also includes policies that contribute to a health promoting environment, such as a well-functioning transportation system, family-friendly labour policies that permit flexi-time and working from home, and the regulation of the commercial environment, including digital food marketing.

## Supporting information

Vaughan et al. supplementary material Vaughan et al. supplementary material

## Supplementary material

For supplementary material accompanying this paper visit https://doi.org/10.1017/S136898002400020X

## References

[1] Swinburn BA , Kraak VI , Allender S et al. (2014) The global syndemic of obesity, undernutrition, and climate change: the lancet commission report. Lancet 393, 791–846. 10.1016/S0140-6736(18)32822-8 30700377

[2] Bates B , Collins D , Cox L et al. (2019) National Diet and Nutrition Survey: Years 1 to 9 of the Rolling Programme (2008/2009–2016/2017) - Time Trend and Income Analyses. London: Public Health England/Food Standards Agency.

[3] Költő A , Gavin A , Molcho M et al. (2020) The Irish Health Behaviour in School-Aged Children (HBSC) Study 2018. Dublin, Ireland: Health Promotion Research Centre, University of Galway and Department of Health, Government of Ireland.

[4] Farrell C , McAvoy H , Wilde J et al. (2008) Tackling Health Inequalities: An all-Ireland Approach to Social Determinants. Dublin: Combat Poverty Agency/Institute of Public Health in Ireland.

[5] Pooler JA , Hartline-Grafton H , DeBor M et al. (2019) Food insecurity: a key social determinant of health for older adults. J Am Geriatr Soc J 67, 421–4. 10.1111/jgs.15736 PMC6816803 30586154

[6] Gundersen C & Ziliak JP (2015) Food insecurity and health outcomes. Health Aff 34, 1830–9. 10.1377/hlthaff.2015.0645 26526240

[7] Gibson R , D’Annibale M , Oliver N et al. (2023) Exploration of the individual, social and environmental factors influencing dietary behaviour in shift workers with type 2 diabetes working in UK healthcare—the shift-diabetes study: a qualitative study using the theoretical domains framework. Diabet Med. Published online: 15 July 2023. doi: 10.1111/jhn.13198. 37452826

[8] Gilmore AB , Fabbri A , Baum F et al. (2023) Defining and conceptualising the commercial determinants of health. Lancet 401, 1194–213. 36966782 10.1016/S0140-6736(23)00013-2

[9] Alkerwi A , Vernier C , Sauvageot N et al. (2015) Demographic and socioeconomic disparity in nutrition: application of a novel correlated component regression approach. BMJ Open 5, e006814-e. 10.1136/bmjopen-2014-006814 PMC4431064 25967988

[10] Monterrosa EC , Frongillo EA , Drewnowski A et al. (2020) Sociocultural influences on food choices and implications for sustainable healthy diets. Food Nutr Bull 41, 59S–73S. 33356592 10.1177/0379572120975874

[11] Mozaffarian D (2016) Dietary and policy priorities for cardiovascular disease, diabetes, and obesity: a comprehensive review. Circulation 133, 187–225. 26746178 10.1161/CIRCULATIONAHA.115.018585 PMC4814348

[12] Bukman AJ , Ronteltap A & Lebrun M (2020) Interpersonal determinants of eating behaviours in Dutch older adults living independently: a qualitative study. BMC Nutr 6, 55. 33292680 10.1186/s40795-020-00383-2 PMC7656669

[13] Marcone MF , Madan P & Grodzinski B (2020) An overview of the sociological and environmental factors influencing eating food behavior in Canada. Front Nutr 7, 77. Published online: 3 June 2020. doi:10.3389/fnut.2020.00077. 32582753 PMC7283517

[14] Grzymisławska M , Puch EA , Zawada A et al. (2020) Do nutritional behaviors depend on biological sex and cultural gender?. Adv Clin Exp Med 29, 165–72. 32017478 10.17219/acem/111817

[15] Serrano-Gonzalez M , Herting MM , Lim S-L et al. (2021) Developmental changes in food perception and preference. Front Psychol. Published online 18 May 2021. doi: 10.3389/fpsyg.2021.654200. PMC8168465 34084148

[16] Baek YJ , Paik HY & Shim JE (2014) Association between family structure and food group intake in children. Nutr Res Pract 8, 463–8. 25110568 10.4162/nrp.2014.8.4.463 PMC4122720

[17] Walsh A & Nelson R (2010) The link between diet and health: an exploratory study of adolescents in Northern Ireland using foodmaps. Int J Consum Stud 34, 190–5.

[18] Fulkerson JA , Friend S , Horning M et al. (2018) Family home food environment and nutrition-related parent and child personal and behavioral outcomes of the healthy home offerings via the mealtime environment (HOME) plus program: a randomized controlled trial. J Acad Nutr Diet 118, 240–51. 28578900 10.1016/j.jand.2017.04.006 PMC5711643

[19] Rahill S , Kennedy A , Walton J et al. (2019) The factors associated with food fussiness in Irish school-aged children. Public Health Nutr 22, 164–74. 30404668 10.1017/S1368980018002835 PMC10260580

[20] Burgess-Champoux TL , Larson N , Neumark-Sztainer D et al. (2009) Are family meal patterns associated with overall diet quality during the transition from early to middle adolescence?. J Nutr Educ Behav 41, 79–86. 19304252 10.1016/j.jneb.2008.03.113

[21] Berge JM , Wall M , Hsueh TF et al. (2015) The protective role of family meals for youth obesity: 10-year longitudinal associations. J Pediatr 166, 296–301. 25266343 10.1016/j.jpeds.2014.08.030 PMC4308550

[22] Harrison ME , Norris ML , Obeid N et al. (2015) Systematic review of the effects of family meal frequency on psychosocial outcomes in youth. Can Fam Physician 61, e96–106. 25676655 PMC4325878

[23] Food Poverty Working Group (2022) Food poverty: Government Programmes, Schemes and Supports. Dublin: Department of Social Protection.

[24] Department of Communities (2021) Family Resources Survey: Northern Ireland 2019/20. Belfast: Department of Communities.

[25] Safefood (2023) What is the cost of a healthy food basket in Ireland in 2022? Dublin: Safefood; available at https://www.safefood.net/Safefood/media/Safefood/PDFs/Food-basket-ROI-Report.pdf (accessed January 2024).

[26] Devereux S , Béné C & Hoddinott J (2020) Conceptualising COVID-19’s impacts on household food security. Food Security 12, 769–72. 32837651 10.1007/s12571-020-01085-0 PMC7358330

[27] Porter JR , Xie L , Challinor AJ et al. (2014) Food security and food production systems. In Climate Change 2014: Impacts, Adaptation, and Vulnerability. Part A: Global and Sectoral Aspects. Contribution of Working Group II to the Fifth Assessment Report of the Intergovernmental Panel on Climate Change, pp. 485–533 [ CB Field , VR Barros , DJ Dokken , KJ Mach , MD Mastrandrea , TE Bilir , M Chatterjee , KL Ebi , YO Estrada , RC Genova , B Girma , ES Kissel , AN Levy , S MacCracken , PR Mastrandrea , and LL White , editors]. Cambridge, United Kingdom and New York, NY, USA: Cambridge University Press.

[28] Barnardos/Amarach (2022) Food poverty: The impact on Vulnerable Children and Families. Dublin: Barnardos.

[29] The Trussell Trust (2022) *End of year stats 2022*; available at https://www.trusselltrust.org/news-and-blog/latest-stats/end-year-stats/#total (accessed January 2024).

[30] Safefood (2022) Exploring the world of food: The perspective of families with children. Dublin: Safefood; available at https://www.safefood.net/worldoffood (accessed January 2024).

[31] Ravikumar D , Spyreli E , Woodside J et al. (2022) Parental perceptions of the food environment and their influence on food decisions among low-income families: a rapid review of qualitative evidence. BMC Public Health 22, 9. 34983469 10.1186/s12889-021-12414-z PMC8727174

[32] Staley K (2015) ‘Is it worth doing?’ Measuring the impact of patient and public involvement in research. Res Involv Engag 1, 6. 10.1186/s40900-015-0008-5 PMC5598089 29062495

[33] Mills S , White M , Wrieden W et al. (2017) Home food preparation practices, experiences and perceptions: a qualitative interview study with photo-elicitation. PLoS One 12, e0182842. 28854196 10.1371/journal.pone.0182842 PMC5576640

[34] Reitz T (2022) Back to the drawing board: creative mapping methods for inclusion and connection. In Co-Creativity and Engaged Scholarship: Transformative Methods in Social Sustainability Research, pp. 323–55 [ A Franklin , editor]. Cham: Springer International Publishing.

[35] Schrecker T (2016) ‘Neoliberal epidemics’ and public health: sometimes the world is less complicated than it appears. Crit Public Health 26, 477–80.

[36] Hsieh H-F & Shannon SE (2005) Three approaches to qualitative content analysis. Qual Health Res 15, 1277–88. 16204405 10.1177/1049732305276687

[37] Bronfenbrenner U (2005) Ecological systems theory (1992). In Making Human Beings Human: Bioecological Perspectives on Human Development, pp. 106–173 [ U Bronfenbrenner , editor]. Thousand Oaks, CA: Sage Publications.

[38] Holford AR (2020) Impact of the Universal Infant Free School Meal Policy, Essex, UK: Institute for Social and Economic Research, University of Essex.

[39] Hatzenbuehler ML , Phelan JC & Link BG (2013) Stigma as a fundamental cause of population health inequalities. Am J Public Health 103, 813–21. 23488505 10.2105/AJPH.2012.301069 PMC3682466

[40] Himmelgreen D , Romero-Daza N , Heuer J et al. (2022) Using syndemic theory to understand food insecurity and diet-related chronic diseases. Soc Sci Med 295, 113–124. 10.1016/j.socscimed.2020.113124 32586635

[41] Shaw SC , Ntani G , Baird J et al. (2020) A systematic review of the influences of food store product placement on dietary-related outcomes. Nutr Rev 78, 1030–45. 32483615 10.1093/nutrit/nuaa024 PMC7666915

[42] Seguin R , Connor L , Nelson M et al. (2014) Understanding barriers and facilitators to healthy eating and active living in rural communities. J Nutr Metab 2014, 146502. 25574386 10.1155/2014/146502 PMC4276670

[43] Balaban DC , Szambolics J & Chirică M (2022) Parasocial relations and social media influencers’ persuasive power. Exploring the moderating role of product involvement. Acta Psychologica 230, 103731. 36057200 10.1016/j.actpsy.2022.103731

[44] Xiang L , Zheng X , Lee MKO et al. (2016) Exploring consumers’ impulse buying behavior on social commerce platform: The role of parasocial interaction. Int J Inf Manag 36, 333–347.

[45] Johnson BK , Potocki B & Veldhuis J (2019) Is that my friend or an advert? The effectiveness of instagram native advertisements posing as social posts. Journal of Computer-Mediated Communication 24, 108–125.

[46] Santoso I , Wright M , Trinh G et al. (2020) Is digital advertising effective under conditions of low attention?. J Market Manage 36, 1707–30.

[47] Brown H , Mills S & Albani V (2022) Socioeconomic risks of food insecurity during the Covid-19 pandemic in the UK: findings from the understanding society covid survey. BMC Publ Health 22, 590. 10.1186/s12889-022-12964-w PMC8960206 35346131

[48] Papaspanos N (2021) Effects of COVID-19 home confinement on eating behaviour and physical activity: results of the ECLB-COVID19 international online survey. Kompass Nutrition & Dietetics 1, 19–21. 10.3390/nu12061583 PMC7352706 32481594

[49] Larson NI , Perry CL , Story M et al. (2006) Food preparation by young adults is associated with better diet quality. J Am Diet Assoc 106, 2001–7. 17126631 10.1016/j.jada.2006.09.008

[50] Hillesund ER , Sagedal LR , Bere E et al. (2021) Family meal participation is associated with dietary intake among 12-month-olds in Southern Norway. BMC Pediatr 21, 128. 33722218 10.1186/s12887-021-02591-6 PMC7958408

