# Supplementary material for: Exploring the world of food with families: perspectives of low-income families on factors influencing their food choices
Source: Public Health Nutr. 2024 Jan 18;27(1):e53. doi: 10.1017/S136898002400020X (PMC10882528; doi:10.1017/S136898002400020X)
Supplement: Vaughan et al. supplementary material [file S136898002400020Xsup001.docx]

**Sample Topic Guide for Photovoice**

**Introduction**

- Thank you very much for taking the time to take these photos. Your participation in this project is highly valued and greatly appreciated
- This interview is to allow us to discuss the photos taken and for you to give any background information to the photos
- I will ask some questions to guide the interview but this session is about your thoughts and insights
- Is it okay for me to switch on the audio recording device now?

**Key questions for *each* photograph:**

Describe your photo?

What is happening in this photo?

Why did you take a photo of this?

What does this picture tell us about your life?

Additional questions:

Would you take this picture prior to the COVID lockdown?

If not, why?

If yes, would there be any differences?

**Closing Questions**

- Is/are your child/ren eligible for free school meals?

If yes, have you received any alternatives while the schools are closed during lockdown (i.e. the weekly vouchers)? What are these alternatives and how useful did you find them?

- Do you have anything you would like to add about any of your photos, the project or your personal experience of planning, shopping and preparing food for your family?
- Are you happy to finish the interview?

**Thank you very much for your time**

**Topic Guide for Mapping Exercises**

**Introduction**

Thank you for creating this map of your food environment. The reason I asked you to do this was so that we can get a sense of the factors that affect your food purchasing habits, choices and meal planning. I would like to ask you some questions about your map so that I can try understand the thought process behind it.

**Questions**

- Could you tell me about the map you have drawn?
- What food outlets such as shops and restaurants do you pass by?
- Do these food outlets influence your food planning or decisions?
- What shops do you use – why do you use these shops?
- Do these shops affect how you plan your meals?
- This landmark is interesting – can you describe it for me?
- Are there bus routes or schools along the way?
- Do you now use any shops or food outlets that you didn’t use before lockdown? Similarly, are there any shops or food outlets that you don’t use now due to lockdown?
- Is there anything else you would like to add about your map?

**Thank you very much for your time**
